# SrrB, a Pseudo-Receptor Protein, Acts as a Negative Regulator for Lankacidin and Lankamycin Production in *Streptomyces rochei*

**DOI:** 10.3389/fmicb.2020.01089

**Published:** 2020-06-09

**Authors:** Yuya Misaki, Shouji Yamamoto, Toshihiro Suzuki, Miyuki Iwakuni, Hiroaki Sasaki, Yuzuru Takahashi, Kuninobu Inada, Haruyasu Kinashi, Kenji Arakawa

**Affiliations:** ^1^Unit of Biotechnology, Graduate School of Integrated Sciences for Life, Hiroshima University, Higashi-Hiroshima, Japan; ^2^Department of Molecular Biotechnology, Graduate School of Advanced Sciences of Matter, Hiroshima University, Higashi-Hiroshima, Japan; ^3^Natural Science Center for Basic Research and Development, Hiroshima University, Higashi-Hiroshima, Japan

**Keywords:** *Streptomyces*, regulatory cascade, pseudo-receptor, antibiotic, biosynthesis

## Abstract

*Streptomyces rochei* 7434AN4, a producer of lankacidin (LC) and lankamycin (LM), carries many regulatory genes including a biosynthesis gene for signaling molecules SRBs (*srrX*), an SRB receptor gene (*srrA*), and a SARP (*Streptomyces* antibiotic regulatory protein) family activator gene (*srrY*). Our previous study revealed that the main regulatory cascade goes from *srrX* through *srrA* to *srrY*, leading to LC production, whereas *srrY* further regulates a second SARP gene *srrZ* to synthesize LM. In this study we extensively investigated the function of *srrB*, a pseudo-receptor gene, by analyzing antibiotic production and transcription. Metabolite analysis showed that the *srrB* mutation increased both LC and LM production over four-folds. Transcription, gel shift, and DNase I footprinting experiments revealed that *srrB* and *srrY* are expressed under the SRB/SrrA regulatory system, and at the later stage, SrrB represses *srrY* expression by binding to the promoter region of *srrY*. These findings confirmed that SrrB acts as a negative regulator of the activator gene *srrY* to control LC and LM production at the later stage of fermentation in *S. rochei*.

## Introduction

**Figure 1:**
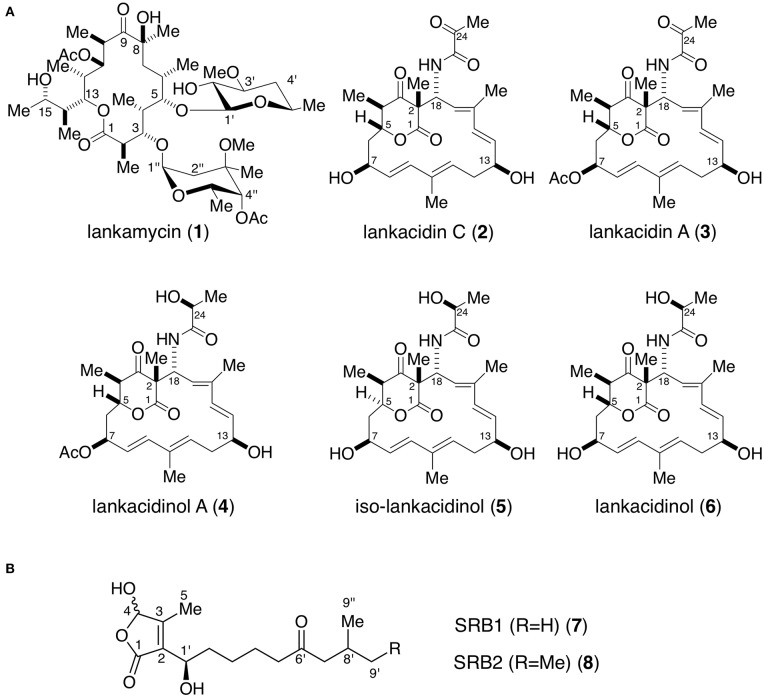
Chemical structures of antibiotics **(A)** and signaling molecules **(B)** produced in *Streptomyces rochei*. **(A)** Antibiotics lankamycin (**1**), lankacidin C (**2**), lankacidin A (**3**), lankacidinol A (**4**), iso-lankacidinol (**5**), and lankacidinol (**6**). **(B)** Signaling molecules SRB1 (**7**) and SRB2 (**8**). Me, methyl; Ac, acetyl.

Secondary metabolites production is strictly controlled by small diffusible signaling molecules that constitute signaling-molecule/receptor regulatory systems in *Streptomyces* species (Bibb, 2005; Takano, 2006; Horinouchi and Beppu, 2007; Martín and Liras, 2019). The most-studied signaling-molecule/receptor system is A-factor/ArpA in *Streptomyces griseus* for streptomycin and grixazone production (Ohnishi et al., 1999, 2005). In the absence of A-factor, ArpA protein specifically binds to the promoter region of the target activator gene *adpA* and represses its transcription. When A-factor reaches a critical concentration, A-factor/ArpA complex dissociates from the promoter region of *adpA*, leading to the onset of *adpA* transcription. Then, the gene product of *adpA* binds to its targets (AdpA-regulons) to activate streptomycin and grixazone production and morphological differentiation (Ohnishi et al., 1999, 2005). Gene sets involved in the signaling-molecule-dependent regulatory pathways for

secondary metabolite production are listed in Table 1 (Arakawa, 2018; Xu and Yang, 2019); e.g., streptomycin and grixazone production in *S. griseus* (Hara and Beppu, 1982; Onaka et al., 1995; Ohnishi et al., 1999, 2005), lankamycin (LM) and lankacidin (LC) (Figure 1) in *Streptomyces rochei* (Arakawa et al., 2007, 2012; Yamamoto et al., 2008; Suzuki et al., 2010), tylosin in *Streptomyces fradiae* (Bate et al., 1999, 2002; Stratigopoulos and Cundliffe, 2002), coelimycin P-1 in *Streptomyces coelicolor* (Takano et al., 2001, 2005; Hsiao et al., 2009; Gottelt et al., 2010; Li et al., 2015), actinorhodin and undecylprodigiosin in *Streptomyces coelicolor* (Xu et al., 2010; Wang et al., 2011), virginiamycin in *Streptomyces virginiae* (Kondo et al., 1989; Kinoshita et al., 1997; Kawauchi et al., 2000), jadomycin in *Streptomyces venezuelae* (Yang et al., 1995; Wang and Vining, 2003; Wang et al., 2009; Xu et al., 2010; Zou et al., 2014), kinamycin in *Streptomyces ambofaciens* (Aigle et al., 2005; Bunet et al., 2008, 2011), and avermectin in *Streptomyces avermitilis* (Kitani et al., 2011; Wang J. B., et al., 2014; Zhu et al., 2016).

**Table 1:** Gene sets of signaling molecule(s)/receptor/pseudo-receptor for secondary metabolite production in *Streptomyces* species.

	**Signaling molecule synthesis gene**	**Signaling molecule(s)**	**Receptor gene**	**Pseudo-receptor gene (pI of gene product)**	**Secondary metabolite(s)**
*S. griseus*	*afsA*	A-factor	*arpA*		Streptomycin, grixazone
*S. rochei*	*srrX*	SRB1, SRB2	*srrA*	*srrB* (11.2)	Lankacidin, lankamycin
*S. fradiae*	ND	ND	*tylP*	*tylQ* (6.4)	Tylosin
*S. coelicolor*	*scbA*	SCB1-3	*scbR*	*scbR2* (5.8)	Coelimycin P-1, actinorhodin, undecylprodigiosin
*S. coelicolor*	*mmfL*	MMFs	*mmfR*		Methylenomycin
*S. virginiae*	*barX*	Virginia butanolides	*barA*	*barB* (10.2)	Virginiamycin
*S. venezuelae*	*jadW1*	SVB1	*jadR3*	*jadR2* (7.8)	Jadomycin
*S. lavendulae*	*farA*	IM-2	*farR*	*farR2* (9.7)	Showdomycin
*S. ambofaciens*	ND	ND	*alpZ*	*alpW* (11.6)	Kinamycins
*S. avermitilis*	*aco*	Avenolide	*avaR1*	*avaR2* (9.6)	Avermectins

*ND, Not determined*.

*Streptomyces rochei* 7434AN4 produces two structurally unrelated polyketide antibiotics, LM and LC (Figure 1A) and carries three linear plasmids pSLA2-L, -M, and -S (Kinashi et al., 1994). Together with the biosynthetic genes for LM and LC, many regulatory genes including a biosynthetic gene for signaling molecules SRBs (Figure 1B) (*srrX*), six *tetR*-type repressor genes (*srrA, srrB, srrC, srrD, srrE*, and *srrF*), and three SARP (*Streptomyces* antibiotic regulatory protein) family activator genes (*srrY, srrZ*, and *srrW*) are located on giant linear plasmid pSLA2-L (210,614 bp) (Mochizuki et al., 2003). Our group revealed that SRBs/SrrA complex dissociates from the promoter region of *srrY*, leading to the activation of LC production (Yamamoto et al., 2008), whereas the gene product of *srrY* further activates a second activator gene *srrZ* to produce LM (Suzuki et al., 2010). In addition, mutation of an additional receptor gene *srrB* greatly increased the production of both LC and LM (Arakawa et al., 2007). This finding suggested that *srrB* negatively regulates LC and LM production, however, its functional role has not been clarified.

In this study, we extensively investigated the function of *srrB* by analyzing antibiotic production and transcription, the results of which indicated that SrrB acts as a negative regulator by binding to the promoter region of the activator gene *srrY* to control LC and LM production at the later stage of fermentation in *S. rochei*.

## Materials and Methods

### Bacterial Strains and DNA Manipulation

*S. rochei* strain 51252 carrying only the linear plasmid pSLA2-L was used as the parent strain (Kinashi et al., 1994). All strains, plasmids, and oligonucleotides used in this study are listed in Table 2. *Streptomyces* strains were grown in YM medium (0.4% yeast extract, 1.0% malt extract, 0.4% D-glucose, pH 7.3) for antibiotic production and RNA isolation. *Escherichia coli* strains were grown in Luria-Bertani (LB) medium supplemented with ampicillin (100 μg/ml), apramycin (50 μg/ml), and/or chloramphenicol (25 μg/ml) when necessary. For protoplasts preparation, *Streptomyces* strains were grown in YEME medium (Kieser et al., 2000). Protoplasts were regenerated on R1M plates (Zhang et al., 1997). DNA manipulations for *E*. *coli* (Sambrook et al., 1989) and *Streptomyces* (Kieser et al., 2000) were performed according to the standard protocols. PCR amplification was done on a 2720 Thermal Cycler (Thermo Fisher Scientific, Waltham, MA, USA) with KOD-Plus- DNA polymerase (Toyobo, Osaka, Japan).

**Table 2:** Bacterial strains, plasmids, and oligonucleotides used in this study.

**Strains/plasmids/oligonucleotides**	**Description**	**Source/References**
**BACTERIAL STRAINS**		
*Streptomyces rochei* 7434AN4	Wild type (pSLA2-L, M, S)	Kinashi et al., 1994
*Streptomyces rochei* 51252	Ultraviolet irradiation of 7434AN4 (pSLA2-L)	Kinashi et al., 1994
*Streptomyces rochei* KA07	*in frame* deletion of *srrB* in 51252 (*ΔsrrB*)	Arakawa et al., 2007
*Streptomyces rochei* KA12	*in frame* deletion of *srrA* in 51252 (*ΔsrrA*)	Arakawa et al., 2007
*Streptomyces rochei* TS03	*in frame* deletion of *srrA* in KA07 (*ΔsrrAB*)	This study
*Streptomyces rochei* KA61	*in frame* deletion of *srrY* in 51252 (*ΔsrrY*)	Yamamoto et al., 2008
*Streptomyces rochei* KA64	*in frame* deletion of *srrY* in KA07 (*ΔsrrBY*)	This study
*Streptomyces rochei* KA20	*kan*::*srrX* in KA07 (*ΔsrrXB*)	Arakawa et al., 2012
*Escherichia coli* XL1-Blue	*recA1 endA1 gyrA96 thi-1 hsdR17 supE44 relA1 lac* F' *proAB lacIqZ*Δ[*M15* Tn10 (Tet^r^)]	Stratagene
*Escherichia coli* BL21(DE3)pLysS	F' *ompT hsdS_*B*_* (r${}_{\text{B}}^{-}$ m${}_{\text{B}}^{-}$) *gal dcm* (DE3) pLysS(Cam^r^)	Novagen
**PLASMIDS**		
Cosmid A8	38.9-kb pSLA2-L DNA (nt 106,868-145,771) cloned into SuperCos-1 at *Bam*HI site	Mochizuki et al., 2003
pKAR3004	4.3 kb *Pst*I-*Eco*47III fragment containing *srrB* in pUC19	(Arakawa et al., 2007)
pKAR3014	3.0 kb *Nco*I-*Sac*I fragment deleted 207-bp *Bsp*EI fragment from *srrA*	Arakawa et al., 2007
pKAR3055	1.5 kb EcoRI-PstI fragment deleted 267-bp *Pvu*II fragment from *srrY*	(Yamamoto et al., 2008)
pKAR3036	1.0 kb *Bgl*II-*EcoI*RI fragment containing *srrB* in pET32b(+)	This study
pKAR4002	9.2 kb *Pst*I fragment containing *srrY* in pUC19	(Yamamoto et al., 2008)
pUC19	Cloning vector; *amp*	Takara
pET32b(+)	T7 expression vector for histidine-tagging, *amp*	Novagen
pIJ8600	Integrative *E. coli-Streptomyces* shuttle vector, inducible *tipA* promoter, *apr, tsr*	Sun et al., 1999
pKAR3065	0.68 kb *Nde*I-*Xba*I PCR fragment containing srrB in pIJ8600	This study
***OLIGONUCLEOTIDE*****S (5′-3′)**		
KAR7903OE	CGCAGATCTACATATGGCCATGCAGGAACGT	This study
KAR7902OE	CTAGAATTCGTACAGCTCGGCCACCATGGC	This study
SRRBf3	ACCCGCACGGCCCGTACATC	This study
SRRBr3	GTACCCCTCTTCCGCGAACA	This study
SRRYf2	GGCGTCGTCTGCCTGCTGCC	Yamamoto et al., 2008
SRRYr2	ATATCCGCCGGGGGCGGTGG	Yamamoto et al., 2008
SRRYf4	CTCCCCTTGTCGTCGTCGAG	This study
SRRYr4	GCGCCCGCGGCGTCACCGAGA	Yamamoto et al., 2008
RT75-F	CAGGTTCTCGTGCGTGCGGTA	This study
RT75-R	GTGCGACGTACAAGCGGGACC	This study
KA82010E	CTAGGATCCGCATATGGCACAGCAGGAAC	This study
SRRAr2B	GGGGGATCCCACCAGCACCGAGGGCACCGC	This study
SRRBf1E	GGGGAATTCGAGCGGTGGAGGACCAGGCCG	This study
SRRBr4	AGGAGCAGTTCCCAGAACGC	This study
16S-357F	CCTACGGGAGGCAGCAG	Turner et al., 1999
16S-907R	CCCCGTCAATTCCTTTGAGTT	Lane 1991
KA-RT079S1	GCGAGACACCGGGAGCCAACTG	This study
KA-RT079AS1	TCGCGGAAGAGGGGTACGTGCC	This study
srrB-8600f1	GAACATATGGCCATGCAGGAA	This study
srrB-8600r1	TGAAGATCTCACTGTCGGGCTG	This study
srrB-GSP1	GCTGCGAACCCAGCTCGAAAC	This study
RT79-R2	CGCCTTCTTGTTCTCGAAGTG	This study
srrB-GSP3	TCCCCGCCGGTGACCGCTCCGTCC	This study

### Construction of Mutant and Plasmid

#### Construction of *srrA* and *srrB* Double Mutant

The target plasmid pKAR3014 that carries in-frame deletion of *srrA* in *E. coli*-*Streptomyces* shuttle vector pRES18 (Ishikawa et al., 1996) was constructed as described previously (Arakawa et al., 2007). Targeted mutagenesis was performed as follows. Plasmid pKAR3014 was transformed into protoplasts of *S. rochei* strain KA07 (*srrB* mutant), and thiostrepton-resistant strains were obtained. Among these transformants, single-crossover strains were selected by Southern hybridization. Some single-crossover colonies were continuously grown in YEME liquid medium to facilitate a second crossover. Finally, thiostrepton sensitive strains were selected as double crossover strains, to obtain a strain TS03 (*srrAB* mutant). Gene disruption was checked by Southern hybridization analysis using DIG DNA Labeling and Detection Kit (Roche Diagnostics GmbH, Mannheim, Germany).

#### Construction of *srrY* and *srrB* Double Mutant

The target plasmid pKAR3055 that carries in-frame deletion of *srrY* in pRES18 was constructed as described previously (Yamamoto et al., 2008). This plasmid was transformed into protoplasts of *S. rochei* strain KA07, and an *srrB*-*srrY* double mutant KA64 was constructed in a similar manipulation as above mentioned.

#### Construction of *in vivo srrB* Expression Plasmid

The *srrB* gene was amplified using cosmid A8 (Mochizuki et al., 2003) and primers, srrB-8600f1 and srrB-8600r1. The resulting PCR product was digested with *Nde*I and *Xba*I and cloned into pIJ8600, an *E. coli*-*Streptomyces* shuttle vector carrying a *tipA* promoter (Sun et al., 1999), to obtain pKAR3065.

This plasmid was introduced into strain 51252, and transformants were cultured for 24 h at 28°C in YM liquid medium with 10 μg/ml apramycin. Thiostrepton (10 μg/ml as final concentration) was added at 24 h to induce *srrB* expression. After cultivation for additional 24 h, the broth filtrate was extracted twice with equal volume of ethyl acetate. The combined organic phase was dried with Na_2_SO_4_, and concentrated *in vacuo* to obtain crude extracts.

#### Construction of *srrB* Overexpression Plasmid in *E. coli*

The *srrB*-coding sequence was PCR amplified using the template cosmid A8 (Mochizuki et al., 2003) and primers, KAR7903OE and KAR7902OE. The amplified fragment was digested with *Bgl*II and *Eco*RI and cloned into pET32b(+), a (His)_6_-tagged expression vector, to obtain pKAR3036.

### Isolation and Analysis of Metabolites

The 48-h cultures of *S. rochei* strains were harvested, and the supernatant was extracted twice with equal volume of ethyl acetate. The crude extracts were purified by Sephadex LH-20 chromatography (1 × 40 cm, GE Healthcare, Chicago, IL) with methanol. Then the fractions containing antibiotics were purified by silica gel chromatography with chloroform-methanol (80:1–10:1, v/v). NMR spectra were recorded on an ECA-500 spectrometer (JEOL, Tokyo, Japan) equipped with a field gradient accessory. Chloroform-*d* and methanol-*d*_4_ were used as solvents. Chemical shifts were recorded in δ value based on the solvent signals (δ_C_ = 77.0 in CDCl_3_, δ_C_ = 49.0 in CD_3_OD, and δ_H_ = 3.30 in residual CH_3_OH) or an internal standard tetramethylsilane (δ_H_ = 0). High resolution ESI-MS spectra were measured by a LTQ Orbitrap XL mass spectrometer (Thermo Fisher Scientific). The ^1^H- and ^13^C-NMR assignments for lankamycin (**1**), lankacidin C (**2**), lankacidin A (**3**), lankacidinol A (**4**), iso-lankacidinol (**5**), and lankacidinol (**6**) have already been reported (Suzuki et al., 2010; Arakawa et al., 2011; Yamamoto et al., 2018).

### SRB Assay

Two strains KA61 (Δ*srrY*) and KA64 (Δ*srrY*Δ*srrB*) were cultured at 28°C for 30 h, and the supernatant (60 ml) was acidified to pH 3 and extracted with equal volume of ethyl acetate twice. The combined organic phase was concentrated in vacuo. Appropriately diluted culture extract (100 μl) was added to the fresh culture (5 ml) of strain KA20, an *srrX*-deficient strain, and cultured at 28°C for 36 h to restore LM and LC production.

### Time-Course Analysis

*S. rochei* strains were grown in YM liquid medium and harvested at various time periods at 12-36 h. Cells were used for measurement of dry cell weight (dcw) and isolation of total RNA, while the culture supernatant was for measurement of antibiotic production.

### Measurement of DCW

Cultures were collected at various time periods and centrifuged at 5,000 rpm for 10 min. The resulting pellet was washed twice with 10.3% sucrose, and then placed in a 60°C dry oven until the weight reaches to a constant value.

### RNA Preparation and Reverse Transcription-PCR (RT-PCR)

*S. rochei* strains were cultured at 28°C in YM liquid medium for various time periods. Total RNAs was extracted from cells with a TRI reagent (Invitrogen, Carlsbad, CA) according to the manufacturer's instructions. Trace amounts of DNA were removed with RNase-free DNase I (Takara, Kyoto, Japan). The concentration of purified RNA was determined by UV absorbance at 260 nm using Ultrospec 3300 pro spectrometer (GE Healthcare). The cDNAs were synthesized using Transcriptor Reverse Transcriptase (Roche Diagnostics). Each reaction mixture contained 1 μg of total RNA and 0.08 A_260_ units random primer. Each mixture was sequentially treated at 85°C for 5 min, at 25°C for 10 min, and 55°C for 45 min for the cDNA synthesis. The 16S rRNA was used as an internal standard (Lane, 1991;Turner et al., 1999).

### 5′ Rapid Amplification of cDNA Ends (5′ RACE)

Transcriptional start site (TSS) of *srrB* was determined using 5′RACE System, Version 2.0 (Invitrogen, Carlsbad, CA, USA). Total RNA was prepared from a 24-h culture sample of parent strain. One microgram of total RNA was converted to the cDNA using specific primer srrB-GSP1, and the resultant was treated with ribonuclease and purified through spin column to afford cDNA. A homopolymeric tail was then added to the 3'-end of cDNA using terminal deoxynucleotidyl transferase and dCTP. PCR was performed with poly C tailed cDNA as a template using abridged anchor primer and inner specific primer RT79-R2. TSS was determined from nucleotide sequence of amplified PCR product using ABI PRISM 310 Genetic Analyzer (Life Technologies, Carlsbad, CA, USA).

### Overexpression and Purification of SrrB Protein

*E. coli* BL21(DE3)pLysS was used as hosts for plasmid pKAR3036. Cells were grown in LB liquid medium supplemented with 100 μg/ml ampicillin and 25 μg/ml chloramphenicol at 37°C to OD_600_ = 0.6 and then were induced with 1 mM isopropyl β-thiogalactopyranoside (IPTG). Cultivation was continued for 12 h at 16°C, and then cells were harvested and disrupted by SONIFER 250 ultrasonic homogenizer (Branson Ultrasonics Corporation, Danbury, CT, USA). The (His)_6_-fusion protein was purified by Ni^2+^-nitrotriacetic acid agarose (Qiagen GmbH, Hilden, Germany) according to the manufacture's protocol. After dialysis with PBS buffer (137 mM NaCl, 8.1mM Na_2_HPO_4_·12H_2_O, 2.68 mM KCl, 1.47 mM KH_2_PO_4_), the (His)_6_-tagged SrrB protein was treated with enterokinase (Novagen, Madison, WI, USA), and the (His)_6_-tag peptide upstream of the N-terminal SrrB was removed by Enterokinase Cleavage Capture Kit (Novagen) according to the manufacture's protocol. The protein was analyzed by SDS-PAGE with 15% polyacrylamide gel. The protein concentration was determined according to the methods of Bradford using Bio-Rad protein assay (Bio-Rad, Hercules, CA, USA) with bovine serum albumin as a standard.

### Preparation of DNA Probes and Gel Shift Assay

The *srrB* probes for gel shift assay were prepared as follows. For preparation of probe B1, a 564-bp DNA fragment containing the upstream region of *srrB* was amplified using pKAR3004 as a template and primers SRRBf3 and SRRBr3 (positions −81 to +483 from TSS of *srrB*; nt 140,677-141,240 of pSLA2-L). For preparation of probe B2, a 386-bp DNA fragment containing the internal region of *srrB* was amplified using pKAR3004 as a template and primers SRRBf1E and SRRBr4 (positions +574 to +959 from TSS of *srrB*; nt 140,201-140,586 of pSLA2-L).

Probe B1 was then 3′ -end labeled with [γ-^32^P]ATP (GE Healthcare) and T4 polynucleotide kinase (Toyobo). The reaction mixture contained the binding buffer (20 mM Tris-HCl [pH 8.0], 100 mM NaCl, 1 mM dithiothreitol, 0.1 mg of bovine serum albumin and 5% glycerol), 0.5 nM labeled DNA and 2 μM SrrA protein. SrrA protein was prepared as reported previously (Yamamoto et al., 2008). When necessary, synthetic SRB1 [(1–′*R*)-isomer; Figure 1B] (Arakawa et al., 2012) was added to the reaction mixture. For competition experiment, unlabeled probes B1 and B2 were used at a final concentration of 200 nM. The reaction mixture was incubated at 26°C for 30 min, and subjected to electrophoresis at room temperature on a native 4.5% polyacrylamide gel in 0.5 × TBE buffer (46 mM Tris base, 46 mM boric acid, 1 mM EDTA). The ^32^P-labeled DNAs were detected by autoradiography.

Preparation of *srrY* probes for gel shift assay was described previously (Yamamoto et al., 2008). To analyze the effect of SRB on the binding of SrrA and SrrB, various concentration of synthetic SRB1 [(1′*R*)-isomer; Figure 1B] (Arakawa et al., 2012) was added to the reaction mixture. In order to evaluate the effect of endogenous metabolites in *S. rochei* and other antibiotics on the binding of SrrB, the following compounds (1 mM) were separately added to the reaction mixture; LC, LM, chlorotetracycline, kanamycin, and ampicillin.

### DNase I Footprinting

The method used for DNase I footprinting analysis for the upstream region of *srrY* was described previously (Yamamoto et al., 2008). For the upstream region of *srrB*, the primer SRRBf3 was 5′-end labeled using [γ-^32^P]ATP (GE Healthcare) and T4 polynucleotide kinase (Toyobo), and then PCR reaction was performed with unlabeled primer SRRBr3 and pKAR3004 as a template to afford a 564-bp product containing the upstream region of *srrB* (positions −81 to +483 from TSS of *srrB*; nt 140,677-141,240 of pSLA2-L). Binding reaction mixture (50 μl) contained 10 nM labeled DNA, 20 mM Tris-HCl (pH8.0), 1 mM MgCl_2_, 100 mM NaCl, 1 mM dithiothreitol, 0.1 mg/ml BSA, 5% glycerol, and various concentrations of SrrA. The binding reaction mixture was incubated for 30 min at 25°C, and then a mixture was treated with DNase I (Roche Diagnostics) solution [1 ng in 50 μl of 5 mM MgCl_2_ and 5 mM CaCl_2_] for 2 minutes at room temperature. The reaction was terminated by 100 μl of phenol-chloroform. The aqueous fraction containing DNAs was precipitated by ethanol and separated on a 5% polyacrylamide gel containing 6 M urea. The labeled DNAs were detected by autoradiography. Sequencing ladders were generated by Maxam-Gilbert sequencing of the labeled DNA used for binding reaction.

### Comparative Sequence Analysis

Alignment of Amino acid sequences of the pseudo-receptors including SrrB was performed by BioEdit version 7.2.5 software (https://bioedit.software.informer.com/) (Hall, 1999) (Figure S1A). Phylogenetic tree was constructed by the neighbor-joining algorithm of MEGA X version 10.1.5 software (Kumar et al., 2018) (Figure S1B).

## Results

### SrrB Acts as a Negative Regulator for Lankacidin and Lankamycin Production

**Figure 2:**
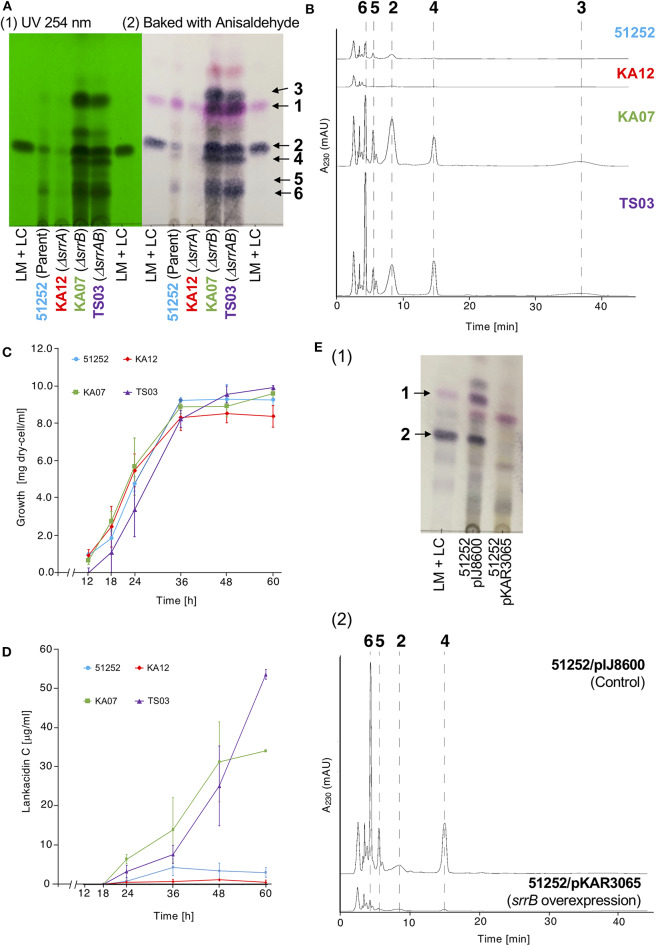
Metabolite profiles and cell growth of four *S. rochei* strains; 51252 (parent), KA12 (Δ*srrA*), KA07 (Δ*srrB*), and TS03 (Δ*srrA-srrB*). **(A)** TLC analysis of the crude extract of *S. rochei* strains. All strains were grown at 28°C for 48 h. The left panel represents the TLC plate under ultraviolet irradiation (254 nm). The right panel represents the TLC plate after baking with anisaldehyde-H_2_SO_4_. TLC plates were developed with chloroform-methanol = 15:1 (v/v). **(B)** HPLC analysis of metabolites produced by *S. rochei* strains. The crude extracts were applied on a COSMOSIL Cholester column (4.6 × 250 mm, Nacalai Tesque) and eluted with a mixture of acetonitrile-10 mM sodium phosphate buffer (pH 8.2) (3:7, v/v) at a flow rate of 1.0 ml/min. **(C)** Time-course growth of *S. rochei* strains. Symbols represent each dry cell weight (DCW; g/l); strain 51252, blue circles and line; strain KA12, red diamonds and line; strain KA07, green squares and line; TS03, purple triangles and line. Results are representative of at least three independent experiments. **(D)** Time-course production of **2** in *S. rochei* strains. Symbols represent each production yield of **2** (μg/ml); strain 51252, blue circles and line; strain KA12, red diamonds and line; strain KA07, green squares and line; TS03, purple triangles and line. Results are representative of at least three independent experiments. **(E)** Effect of *srrB* overexpression on antibiotic production. Thiostrepton (10 μg/ml) was added to the 24-h culture of *S. rochei* 51252 recombinants harboring either pIJ8600 (control) or pKAR3065 (intact *srrB*), and then the cultures were further incubated at 28°C for 24 h. (1) TLC analysis of crude extracts. Crude extracts were separated by TLC [eluent; chloroform-methanol = 15:1 (v/v)] and detected by baking with anisaldehyde-H_2_SO_4_. Lane 1, LM and LC standard; lane 2, recombinant harboring pIJ8600 (control); lane 3, recombinant harboring pKAR3065 (intact *srrB*). (2) HPLC analysis of crude extracts. The crude extracts were applied on a COSMOSIL Cholester column (4.6 x 250 mm, Nacalai Tesque) and eluted with a mixture of acetonitrile-10 mM sodium phosphate buffer (pH 8.2) (3:7, v/v) at a flow rate of 1.0 ml/min.

The gene product of *srrB* belongs to the TetR-type transcriptional regulator family proteins, which contains a helix-turn-helix DNA binding motif at the N-terminal region (Figure S1). We previously reported overproduction of LM and LC in the *srrB* mutant KA07 based on TLC bioautography (Arakawa et al., 2007). In this study, we performed comparative metabolite analysis of the *srrB* mutant and its parent strain 51252. As shown in Figures 2A,B, the *srrB* mutant KA07 accumulated larger amount of compounds **1**-**6** compared with the parent strain 51252. Namely, KA07 produced 6-folds of lankamycin (**1**) (Figure 2A) and 9.9, 25, 4.2, and 5.7-folds of lankacidin C (**2**), lankacidinol A (**4**), iso-lankacidinol (**5**), and lankacidinol (**6**), respectively (Figure 2B). To investigate the effect of SrrA on antibiotic production, we further analyzed two mutants, an *srrA* mutant KA12 and an *srrA*-*srrB* double mutant TS03 (Figure S2). KA12 produced about 40% of metabolites when compared with the parent, while TS03 overproduced metabolites **1**-**6** at the same level with the *srrB* mutant KA07 (Figures 2A,B). These results confirmed the following two aspects; *srrA* mutation causes a slight decrease of the metabolic titer, whereas SrrB acts as a negative regulator for lankacidin and lankamycin production in *S. rochei*.

To determine the role of *srrB* in the regulation of lankacidin and lankamycin production, we further performed time-course analysis of metabolite profile, growth curve, and transcription in the parent and three mutants (KA07, KA12, and TS03) at various time periods. As shown in Figure 2C, all strains grew in a similar proportion, indicating that overproduction in KA07 and TS03 was due to *srrB* mutation but not to cell growth difference. The time-course of antibiotic production was analyzed by the titer of **2**, a major product among lankacidin derivatives (**2**-**6**; Figure 1A). As shown in Figure 2D, **2** was detected after 18 h, and its titer at 48-h growth in the *srrB* deficient strains, KA07 and TS03, were 9.0- and 7.2-times of 51252, respectively, which agrees with the overproduction profiles in KA07 and TS03 in Figures 2A,B. To confirm the negative regulatory property of SrrB *in vivo*, overexpression of SrrB in *S. rochei* was carried out. The intact *srrB* gene was introduced into plasmid pIJ8600, an *E. coli*-*Streptomyces* shuttle plasmid with a thiostrepton-inducible *tipA* promoter, to give pKAR3065. We tested antibiotic production in the *S. rochei* 51252 recombinants containing either the empty vector pIJ8600 or the *srrB* overexpression plasmid (pKAR3065). Compared with the control recombinant *S. rochei* 51252/pIJ8600, the *S. rochei* 51252/pKAR3065 recombinant significantly reduced antibiotic production (Figure 2E). These results clearly indicated that SrrB acts as a negative regulator for antibiotic production in *S. rochei*.

In our preliminary experiment, gel shift assay indicated that SrrA and SrrB could bind to the upstream region of *srrX*, a gene responsible for SRB biosynthesis. This finding suggested that the signaling molecule receptor/pseudo-repressor repress the transcription of *srrX*. Two strains were used to evaluate the comparative yield of SRB; an *srrY* single mutant KA61 and an *srrY*-*srrB* double mutant KA64 (Figure S3), both of which are unable to produce LC or LM due to a mutation on the major activator *srrY*. The yields of SRBs were evaluated by a help of bioassay using an *srrX*-deficient mutant as described previously (Arakawa et al., 2012). One-eighth of the crude extract of KA64 contained an equivalent amount of SRBs to that of KA61 (Figure S4), suggesting that *srrB* negatively controls the titer of SRBs.

### *srrB* and *srrY* Are Expressed Under the SRB/SrrA Regulatory System, and SrrB Then Represses *srrY* Expression at the Later Stage

**Figure 3:**
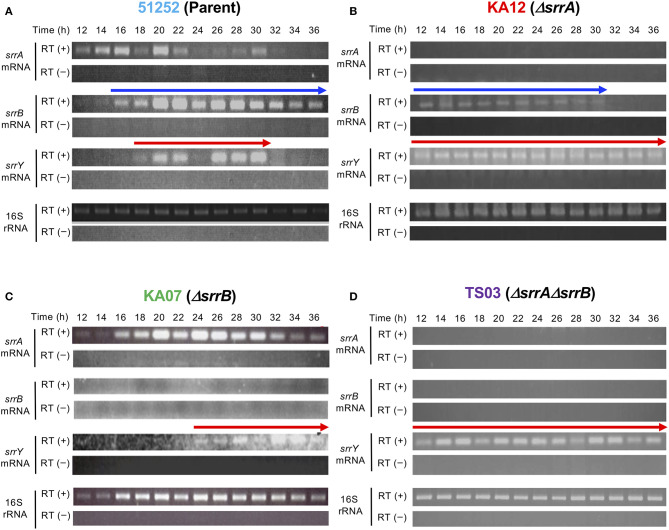
Time-course RT-PCR analysis of strains 51252 **(A)**, KA12 **(B)**, KA07 **(C)**, and TS03 **(D)**. Three upper panels represent *srrA* mRNA, *srrB* mRNA, and *srrY* mRNA. The lowest panels represents 16S rRNA gene as a control. RT (+) indicates the treatment of total RNA with Transcriptor Reverse Transcriptase, while RT (–) dues no treatment of transcriptase. Red arrow indicates transcription of *srrY*, while blue arrow does transcription of *srrB*.

To analyze the role of *srrB* in the SRB/SrrA regulatory system in *S. rochei*, we performed comparative transcriptional analysis of the selected regulatory genes in the parent and three mutants (*srrA, srrB*, and *srrA*-*srrB*). Transcription of *srrY* in the parent appeared around 18 h and diminished after 32 h (Figure 3A), while that in the *srrB* mutant KA07 continued until the later stage (Figure 3C). On the other hand, *srrB* transcription in the parent appeared around 16 h and prolonged until the late stage of fermentation (Figure 3A). In the *srrA* mutant KA12, transcription of both *srrY* and *srrB* appeared at 12 h or earlier (Figure 3B), whereas *srrY* transcription in the *srrA-srrB* mutant TS03 was detected through all time periods (12–36 h) (Figure 3D). These findings together with our previous result (Yamamoto et al., 2008) showed that SRB/*srrA* regulatory system controls transcription of both *srrY* and *srrB*, and SrrB represses *srrY* transcription at the later stage.

### SrrA Binds to the Promoter Region of *srrB*

**Figure 4:**
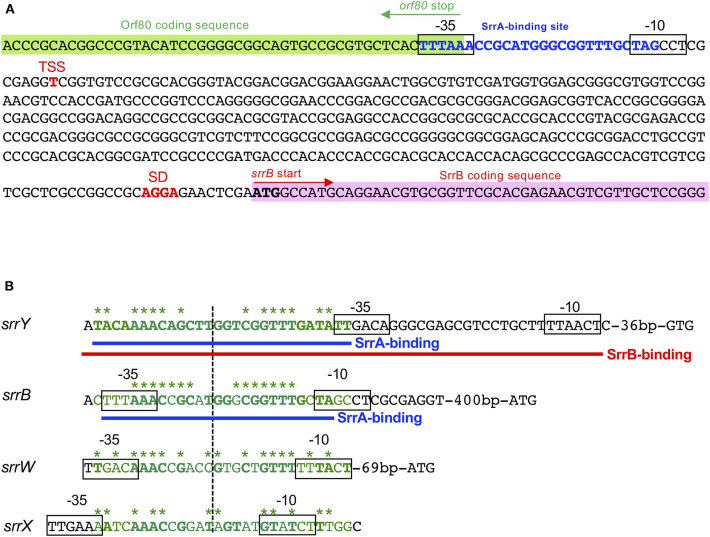
SrrA/SrrB binding sequences. **(A)** Characterization of the upstream region of *srrB*. Putative *srrB* promoter regions (−35 and −10) are boxed. Shine-Dalgarno (SD) sequence and transcriptional start site (TSS) are shown as red boldface letters. SrrA-binding sequence is shown as blue boldface letters. Pink and green highlights indicate SrrB and Orf80 coding sequences, respectively. **(B)** Comparison of the binding sequences for SrrA and SrrB. The confirmed SrrA- and SrrB-binding sequences are shown as blue and red underlines, respectively. Possible SrrA-binding sites at upstream of *srrW* and *srrX* are deduced from sequence data. For comparison of consensus sequence, SrrA-binding sites at the upstream of *srrY* (SrrA-*srrY*) are shown as green. Bases identical with SrrA-*srrY* are shown in boldface letters. Complementary bases are indicated as asterisks. The center of palindromes is shown as a vertical dashed line.

**Figure 5:**
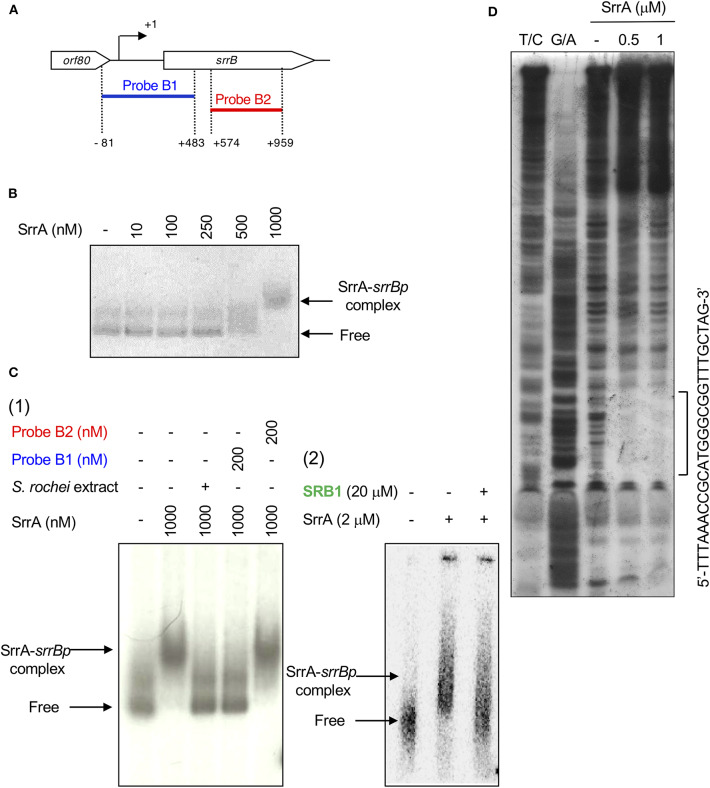
Gel shift assay and DNase I footprinting analysis of SrrA-binding in the upstream region of *srrB*. **(A)** Location of the two probes B1 and B2. The TSS of *srrB* is numbered +1. **(B)** The concentration-dependent binding of SrrA to the upstream promoter region of *srrB* (*srrBp*). The ^32^P-labeled probe B1 (1 nM) was mixed with various concentration of SrrA (0–1,000 nM). **(C)** (1) Competition experiments to investigate specific binding of SrrA to the upstream region of *srrB*. Each reaction mixture contained 0.5 nM ^32^P-labeled probe B1 (lane 1) and 1,000 nM SrrA (lane 2). Then, *S. rochei* culture extract (lane 3), 200 nM unlabeled probe B1 (lane 4) or unlabeled probe B2 (lane 5) was added. (2) Effect of endogenous SRB to investigate specific binding of SrrA to the upstream region of *srrB*. Each reaction mixture contained 0.5 nM ^32^P-labeled probe B1 (lane 1) and 2,000 nM SrrA (lane 2). Then, 20 μM synthetic SRB1 [(1′*R*)-isomer; Figure 1B] (Arakawa et al., 2012) (lane 3) was added. **(D)** DNase I footprinting analysis of SrrA-binding site on the upstream of *srrB*. Probe B1 was end labeled on the non-template strand. Each reaction mixture contained 2 nM labeled DNA and SrrA (0.5 and 1 μM). Sequencing ladders were generated by Maxam-Gilbert sequencing of the labeled probe B1. Capital letters at right side indicate SrrA-binding sequences.

A transcriptional start point (TSS) of *srrB* was determined be 401-bp upstream of its translational start codon by 5′-RACE (Figure 4 and Figure S5). To determine whether SrrA binds to the upstream promoter region of *srrB* (*srrBp*), gel shift assay was performed using ^32^P-labeled probe B1 (nt 140,677-141,240 of pSLA2-L) that contained the upstream region of *srrB* (Figure 5A). SrrA protein was overexpressed in *E. coli* as described previously (Yamamoto et al., 2008). A band shift of probe B1 was observed in the presence of SrrA protein in a concentration-dependent manner (Figure 5B). Competition experiments using unlabeled probes B1 and B2 (nt 140,201-140,585 of pSLA2-L) (Figure 5C) were performed to determine the specific binding of SrrA to the region of probe B1. A band shift disappeared in the large excess of unlabeled probe B1, whereas probe B2 gave no effect on band shift (Figure 5C). Addition of SRB led to dissociation of SrrA from probe B1 (Figure 5C), indicating that the *srrB* transcription is controlled by SRB/SrrA regulatory system.

DNase I footprinting experiment was performed to identify the SrrA binding sequence in the upstream of *srrB*. Positions −36 to −11 of the non-template strand was protected by SrrA (Figure 5D). The protected region overlapped with a possible *srrB* promoter containing a palindromic sequence (asterisks in Figure 4B), whose sequence well-matched with the SrrA-binding sequence of *srrY* (Yamamoto et al., 2008). Taking account of transcriptional analysis above mentioned, SrrA binds to the upstream regions of both *srrB* and *srrY* to repress their transcription at the early growth phase (~16 h).

### SrrB Represses *srrY* Transcription at the Later Stage of Fermentation by Binding to the Promoter Region of *srrY*

**Figure 6:**
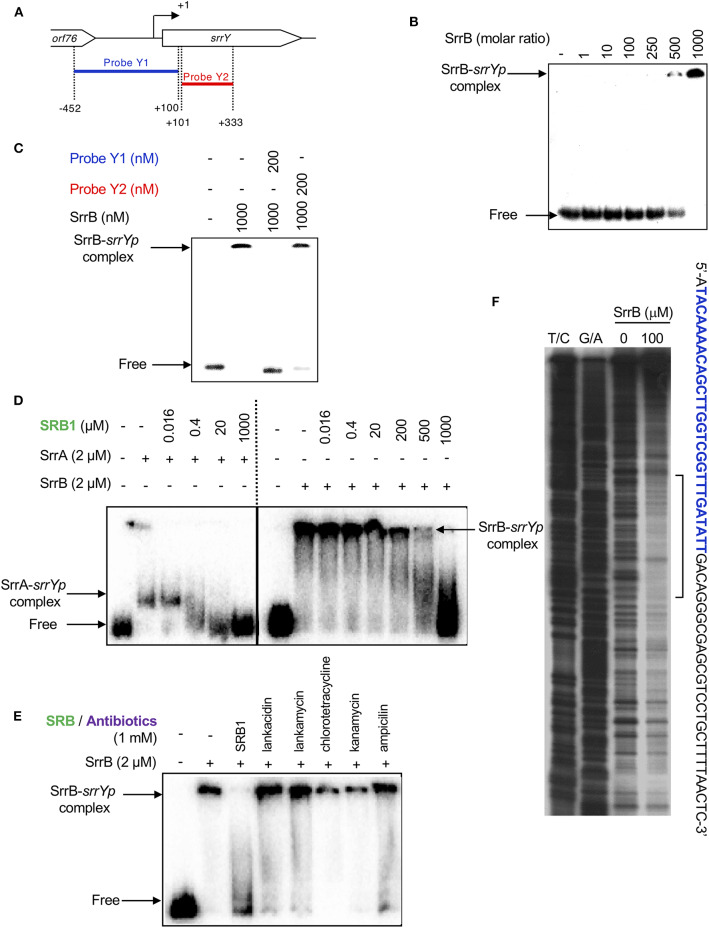
Gel shift assay and DNase I footprinting analysis of SrrB-binding in the upstream region of *srrY*. **(A)** Location of the two probes, probe Y1 and Y2. The description of probe preparation was described previously (Yamamoto et al., 2008). **(B)** The concentration-dependent binding of SrrB to the upstream promoter region of *srrY* (*srrYp*). Labeled probe Y1 (1 nM) was mixed with various concentration of SrrB (0–1,000 nM). **(C)** Competition experiments to investigate specific binding of SrrB to the upstream region of *srrY*. Each reaction mixture contained 1 nM ^32^P-labeled probe Y1 and 1,000 nM SrrB. Then, 200 nM unlabeled probe Y1 (lane 3) or unlabeled probe Y2 (lane 4) was added. **(D)** Effect of SRB1 on the binding of SrrA (Right panel) and SrrB (left panel). Each reaction mixture contained 0.5 nM probe Y1 and 2,000 nM recombinant protein. To the reaction mixture, various concentration of synthetic SRB1 [(1′*R*)-isomer; Figure 1B) (Arakawa et al., 2012) was added. **(E)** Effect of endogenous metabolites in *S. rochei* and other antibiotics on the binding of SrrB. To the same reaction mixture described for panel D, various compounds including SRB1, LC, LM, chlorotetracycline, kanamycin, and ampicillin (each 1 mM) were separately added. **(F)** DNase I footprint analysis of SrrB-binding site on the upstream of *srrY*. Probe Y1 was end labeled on the non-template strand. Each reaction mixture contained 2 nM labeled DNA and SrrB (100 nM). Sequencing ladders were generated by Maxam-Gilbert sequencing of the labeled probe Y1. Capital letters at right side indicate SrrB-binding sequences, among which blue letters indicate SrrA-binding sequences.

To analyze whether SrrB binds to the promoter region of *srrY* (*srrYp*), gel shift assay was performed using probe Y1 (positions −452 to +100 from TSS of *srrY*) (Figure 6A) containing the promoter region of *srrY*, which was constructed previously (Yamamoto et al., 2008). SrrB protein was overexpressed in the *E. coli* BL21(DE3)pLysS/pKAR3036 recombinant with IPTG induction and purified by Ni-NTA agarose (Figure S6). The addition of SrrB protein gave a shifted band of probe Y1 in a concentration-dependent manner (Figure 6B). Competitive experiments (Figure 6C) revealed that SrrB specifically binds to probe Y1, not to probe Y2 (positions +101 to +333 from TSS of *srrY*). The pseudoreceptors hitherto studied are insensitive to endogenous signaling molecules and interact with endogenous antibiotics (Martín and Liras, 2019; Xu and Yang, 2019) (details are described in Discussion Section). We tested the effects of various signaling-molecule/antibiotics on the binding of SrrB to *srrYp* through gel shift analysis by using endogenous signaling molecule SRB, endogenous antibiotics (LC and LM), and other exogenous antibiotics (Figures 6D,E). Dissociation of SrrB from *srrYp* could be caused by SRB, however, a higher concentration of 1 mM was required (500-fold excess against SrrB protein). The sensitivity of SrrB against SRB was 50-fold lower compared with the signaling molecule receptor SrrA (Figure 6D). Dissociation of SrrB from *srrYp* was not caused by endogenous antibiotics LC and LM in *S. rochei* and neither by exogenous antibiotics (chlorotetracycline, kanamycin, ampicillin) at even 1 mM concentration (500-fold excess against SrrB protein) (Figure 6E).

We further performed DNase I footprinting experiment to identify the SrrB binding sequence in the upstream of *srrY*. As shown in Figure 6F, positions −61 to −4 of non-template strand were protected by SrrB. Although SrrB covers larger upstream region (58 bp) than SrrA does (28 bp; blue letters in Figure 6F), both SrrA and SrrB could bind to the promoter region of *srrY* (Figure 4B).

## Discussion

In this study, we revealed that *srrB* acts as a negative regulator by binding to the promoter region of the target gene *srrY* to repress LC and LM production in *S. rochei*. Expression of *srrB* is controlled by SRB/SrrA regulatory system.

TetR-type receptors have a conserved DNA-binding helix-turn-helix motif in the N-terminus and a ligand-binding pocket in the C-terminus (Yu et al., 2010). Particularly, the signaling molecule receptors and the pseudo-receptors constitute one of a major class of TetR-type regulators (Figure S1). The signaling molecule receptors have a helix-turn-helix DNA-binding motif in the N-terminus and a ligand-binding Trp residue in the C-terminus. It is noteworthy that the signaling molecule synthase and its cognate receptor gene pairs usually locate adjacently on the genome (Biarnes-Carrera et al., 2015), which allows us to predict signaling molecule/receptor systems in *Streptomyces* species (Niu et al., 2016). On the other hand, the pseudo-receptors also have a conserved DNA-binding motif like the signaling molecule receptors (Figure S1A), however, their location has no relationship with the signaling molecule synthase genes. Many of them act as negative regulators for antibiotic production; for example, TylQ for tylosin production in *Streptomyces fradiae* (Stratigopoulos and Cundliffe, 2002), BarB for virginiamycin in *Streptomyces virginiae* (Matsuno et al., 2004), ScbR2 for coelimycin P-1 in *Streptomyces coelicolor* (Gottelt et al., 2010), AlpW for orange pigment kinamycin in *Streptomyces ambofaciens* (Bunet et al., 2008), and AvaR2 for avermectin in *Streptomyces avermitilis* (Zhu et al., 2016) (Table 1).

In general, the pseudo-receptors are insensitive to endogenous signaling molecules. BarB has no binding affinity to virginia butanolides in *S. virginiae* (Matsuno et al., 2004). Surprisingly, ScbR2 from *S. coelicolor* does not bind to the signaling molecules SCB1-3 but binds to two endogenous antibiotics, actinorhodin and undecylprodigiosin (Xu et al., 2010), and exogenous antibiotic jadomycin (Wang W., 2014). In *S. venezuelae*, JadR2 binds to endogenous jadomycin and chloramphenicol as ligands, (Xu et al., 2010). Thus, ScbR2 and JadR2 bind to multiple antibiotics, and coordinate their biosynthesis (Xu et al., 2010; Zou et al., 2014). In *S. avermitilis*; AvaR2 binds to the endogenous signaling molecule avenolide, but not to oligomycin and avermectin (Zhu et al., 2016). Its mutational analysis revealed that AvaR2 plays a negative regulatory role in avermectin production and cell growth (Zhu et al., 2016). In *S. rochei*, SrrB-*srrYp* complex was disrupted by endogenous signaling molecule SRB at 1 mM concentration, although its minimum dissociation concentration for SrrB was 50-fold higher than that for SrrA, the SRB receptor. SrrB showed no binding activity to endogenous polyketide antibiotics LM or LC in *S. rochei* and neither to exogenous antibiotics including aromatic polyketide chlorotetracycline, aminoglycoside antibiotic kanamycin, and β-lactam antibiotic ampicillin even at 1 mM concentration (500-fold excess against SrrB). Thus, functions of the pseudo-receptors are variable in *Streptomyces* species.

**Figure 7:**
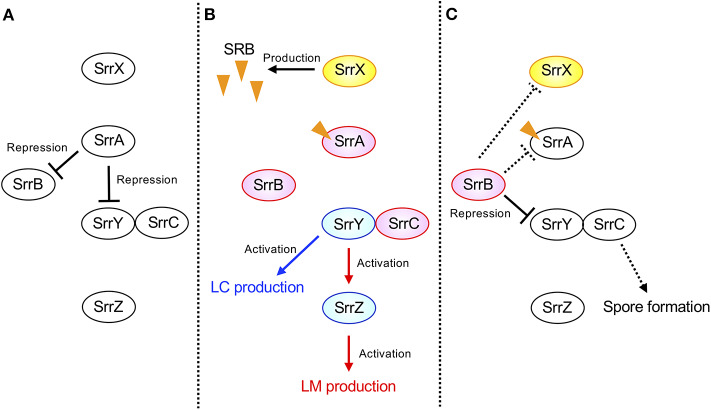
Model of transient *srrY* expression for LM and LC production in *S. rochei*. **(A)** SrrA represses both *srrY* and *srrB* expression in the early stage of growth. **(B)** Dissociation of SrrA from the promoter region of both *srrY* and *srrB* by SRB in middle stage of growth. **(C)** Repression of *srrY* by SrrB in the late stage of growth. Solid lines indicate the confirmed regulatory pathway hitherto. Additional dashed lines were suggested by unpublished results. Orange triangles indicate the signaling molecules SRBs.

The possible regulatory pathway in *S. rochei* is shown in Figure 7. At the early growth phase, SrrA represses transcription of both *srrY* and *srrB* (panel A). When SRB reaches a critical concentration, SrrA-SRB complex dissociates from both promoter regions to induce expression of *srrY* and *srrB* (panel B). Then SrrB represses *srrY* transcription at the later stage fermentation (panel C), suggesting a transient expression of *srrY* by two receptor proteins SrrA and SrrB in *S. rochei*. A similar regulatory pathway was proposed for kinamycin production in *S. ambofaciens* (Bunet et al., 2008) although its ligand has not yet been identified. In the early stage of growth, the signaling molecule receptor AlpZ represses both transcription of *alpV* (an SARP-type activator gene) and *alpW* (a pseudo-receptor gene). When an unidentified ligand interacts with AlpZ, this protein dissociates from the promoter regions in both *alpV* and *alpW*, leading to kinamycin production. At the later stage of fermentation, AlpW represses *alpV* transcription again to cease kinamycin production. Another interesting features in the *S. rochei* regulatory pathway is the presence of *srrY-srrC* cistron (Figure 7). The *srrC* mutant showed no sporulation, suggesting that *srrC* acts as a positive regulator for morphological differentiation (Arakawa et al., 2007). As shown in Figure 7, *srrB* negatively regulates the transcription of both *srrY* and *srrC*, which leads to transient controls for antibiotic production and morphological differentiation, respectively.

The *srrB* mutation increased the titers of antibiotics as well as SRBs. This result well agreed with our preliminary gel shift assay that both SrrA and SrrB bind to the upstream region of SRB biosynthesis gene *srrX* (data not shown). Large excess of SRBs has no effect on antibiotic overexpression in *S. rochei* (Arakawa et al., 2012), hence, exact mechanism of the *srrX* repression by SrrB at the later stage remains to be clarified.

Manipulation of regulatory genes often causes activation of “silent” secondary metabolites (Olano et al., 2008; Zerikly and Challis, 2009; Rutledge and Challis, 2015; Arakawa, 2018). In *S. ambofaciens*, a mutant of the pseudo-receptor gene *alpW* accumulated kinamycin (Bunet et al., 2011). A mutant of the repressor gene *ksbC* accumulated β-carboline compound kitasetaline in *Kitasatospora setae* (Aroonsri et al., 2012). To our surprise, azoxyalkene compound KA57-A accumulated in a triple knockout mutant of *S. rochei* that have mutations on two biosynthetic gene clusters for LC, LM, and *srrB* (Kunitake et al., 2015). The genome sequence of the *S. rochei* chromosome has been determined to be 8.36 Mb in size, and at least 35 secondary metabolites gene clusters are coded on the chromosome (Nindita et al., 2019). Comprehensive mutational analysis on various regulatory genes may lead to activate silent secondary metabolite gene clusters in *S. rochei*, which is in progress in our laboratory. In conclusion, we have extensively characterized the role of the pseudo-receptor SrrB for antibiotic production in *S. rochei*. Further understanding and manipulation of the regulatory system in *Streptomyces* will lead to a natural product discovery with notable biological activities.

## Data Availability Statement

The datasets generated for this study are available on request to the corresponding author.

## Author Contributions

YM, SY, TS, HK, and KA designed the experiments. YM, SY, TS, MI, HS, YT, KI, and KA performed the experiments. YM, SY, TS, MI, HS, and KA analyzed the data. YM, SY, TS, HK, and KA wrote the manuscript with input from all of the authors. All authors approved the final version of the manuscript.

## Conflict of Interest

The authors declare that the research was conducted in the absence of any commercial or financial relationships that could be construed as a potential conflict of interest.
