# Supplementary material for: SrrB, a Pseudo-Receptor Protein, Acts as a Negative Regulator for Lankacidin and Lankamycin Production in Streptomyces rochei
Source: Front Microbiol. 2020 Jun 9;11:1089. doi: 10.3389/fmicb.2020.01089 (PMC7296167; doi:10.3389/fmicb.2020.01089)
Supplement: Supplementary file 1 [file Data_Sheet_1.PDF]

Supplementary Materials

SrrB, a Pseudo-Receptor Protein, Acts as a Negative Regulator for Lankacidin and Lankamycin Production in *Streptomyces rochei*

Yuya Misaki[1,2,†], Shouji Yamamoto[2,†,¶], Toshihiro Suzuki[2,#], Miyuki Iwakuni[2], Hiroaki Sasaki[2], Yuzuru Takahashi[2], Kuninobu Inada[3], Haruyasu Kinashi[2], and Kenji Arakawa[1,2*]

[1]Unit of Biotechnology, Graduate School of Integrated Sciences for Life, and [2]Department of Molecular Biotechnology, Graduate School of Advanced Sciences of Matter, Hiroshima University, 1-3-1 Kagamiyama, Higashi-Hiroshima, Hiroshima 739-8530, Japan. [3]Natural Science Center for Basic Research and Development, Hiroshima University, 1-4-2 Kagamiyama, Higashi-Hiroshima 739-8526, Japan.

**** Correspondence :*** Kenji Arakawa (karakawa@hiroshima-u.ac.jp)

[†] These authors have contributed equally to this work.

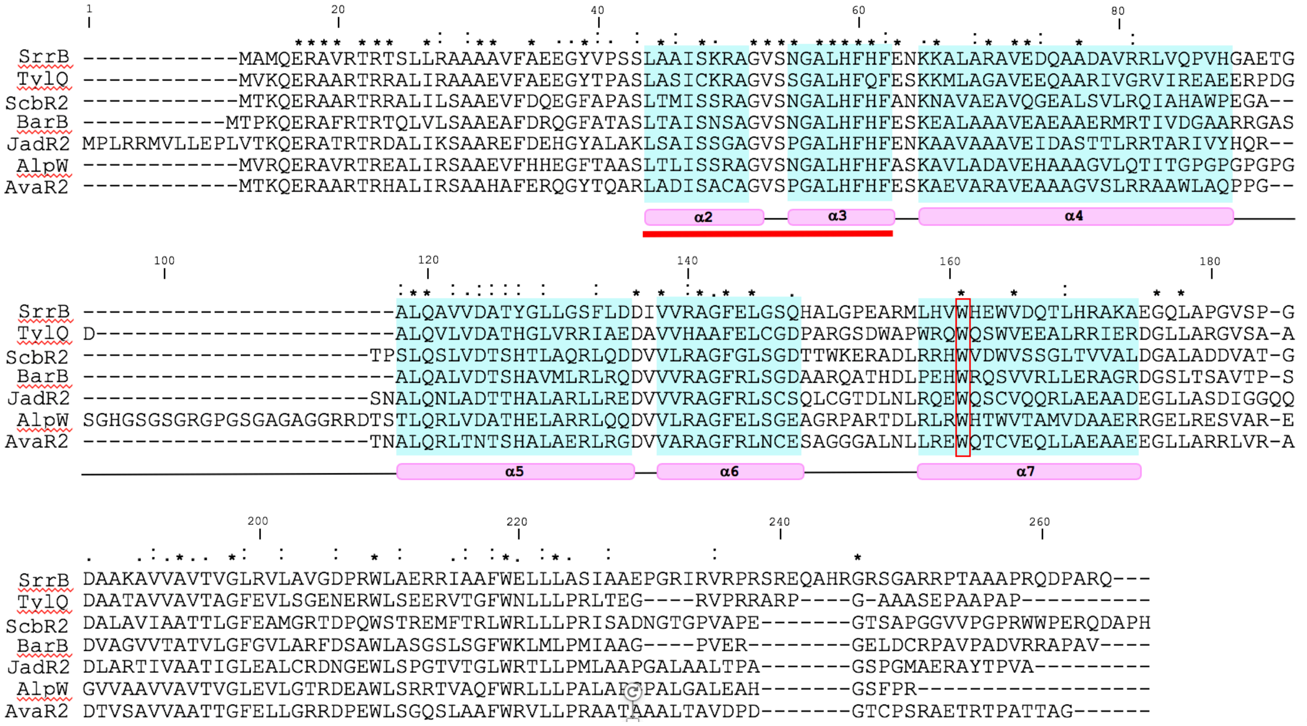

Figure S1. (**A**) Alignment of the deduced amino acid sequences of SrrB and SrrB homologues. This alignment was performed by BioEdit version 7.2.5 software (http://www.mbio.ncsu.edu/BioEdit/bioedit.html) (Hall 1999). Conserved α-helix structures are shaded light blue. Helix-turn-helix motif at the N-terminal is marked as a red underline. Red box indicates an important residue for ligand binding. Highly conserved amino acid residues are marked below the alignment as identical (asterisk), well conserved (colon), or partially conserved (period). The GenBank accession numbers are shown as follows: *S. rochei*, SrrB (NP851501); S. *fradiae*, TylQ (AAD40803); *S. coelicolor* A3(2), ScbR2 (NP630384); *S. virginiae*, BarB (BAA23612); *S. venezuelae*, JadR2 (CCA59258); *S. ambofaciens*, AlpW (CAJ87891); *S. avermitilis*, AvaR2 (BAC71414).

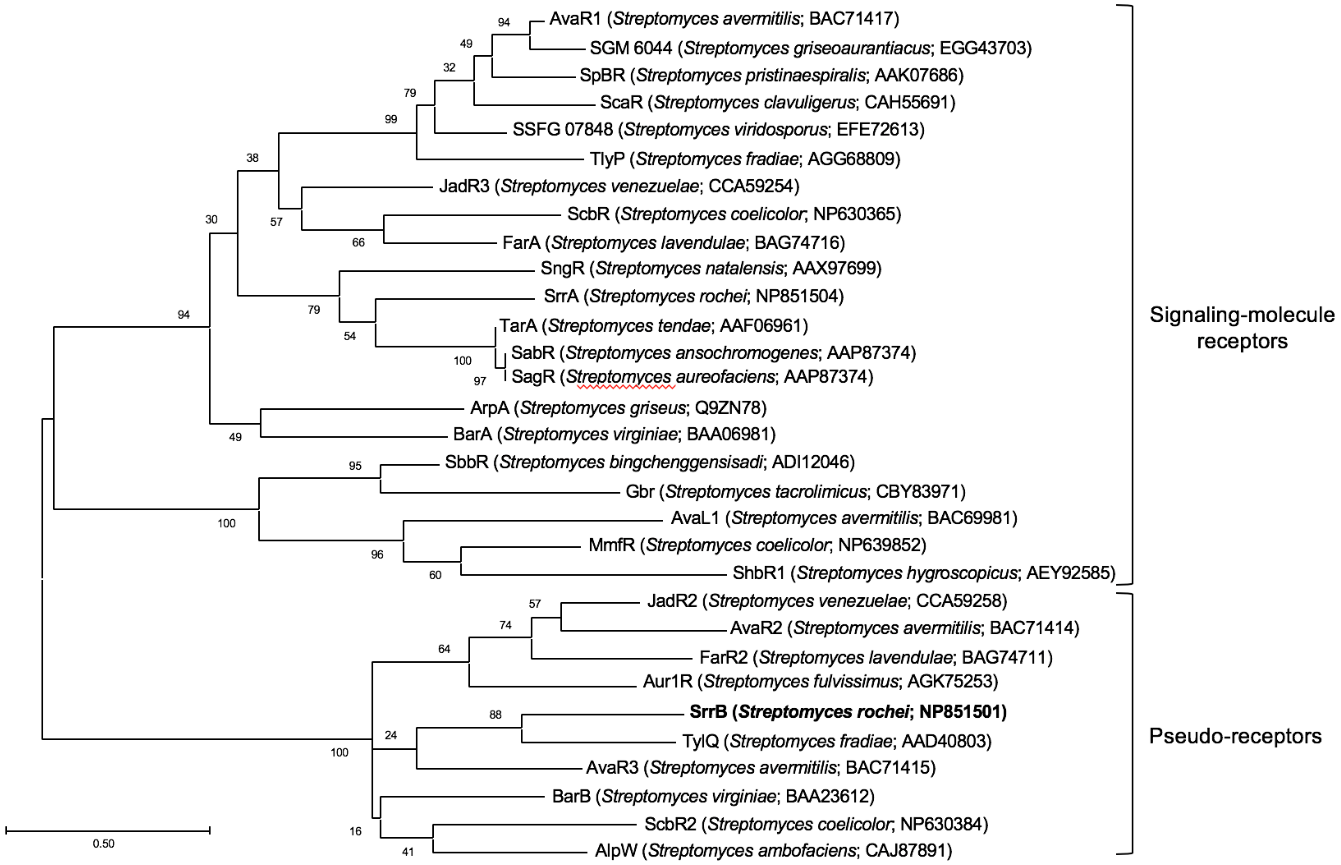

Figure S1. (**B**) Phylogenetic analysis of the signaling-molecule receptors and the pseudo-receptors. Phylogenetic tree was constructed by the neighbor-joining algorithm of MEGA X version 10.1.5 software (Kumar et al. 2018). Bootstrap values, expressed as a percentage of 1000 replications, are given at branching points.

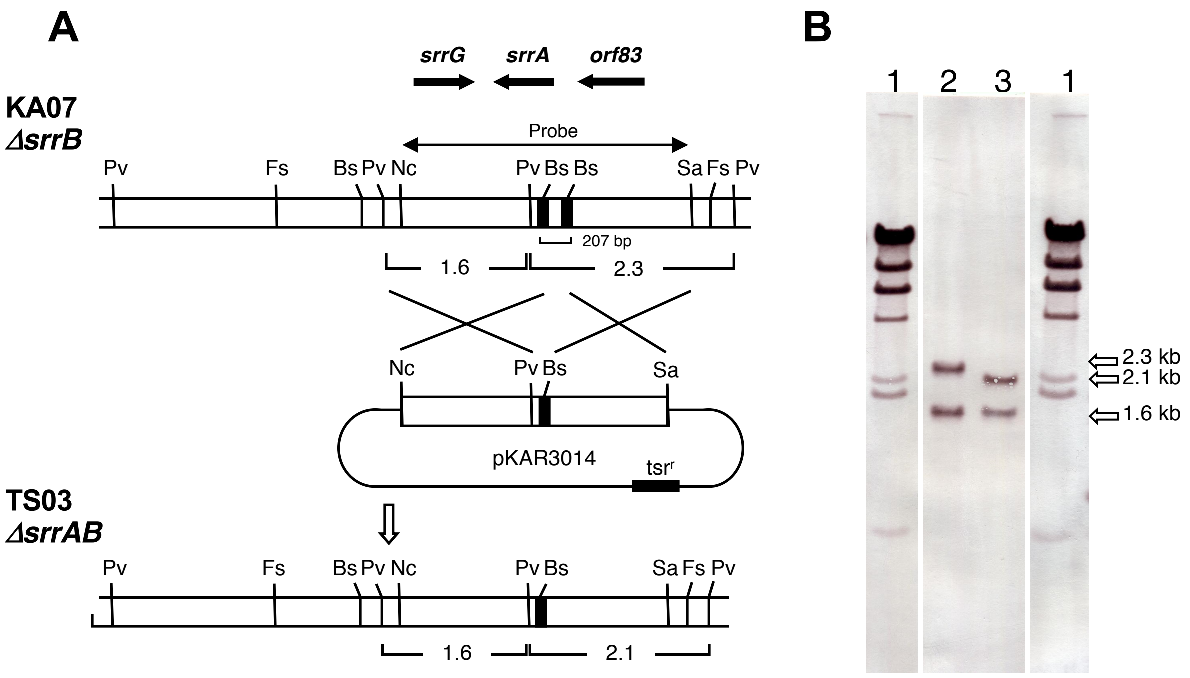

Figure S2. (**A**) Construction of the *ΔsrrAB* mutant TS03. Pv, *Pvu*II; Fs, *Fsp*I; Bs, *Bsp*EI; Nc, *Nco*I; Sa, *Sac*I. (**B**) Southern blot analysis. Lane 1, λ/*Hin*dIII; lane 2, strain 51252 (parent)/ *Pvu*II; lane 3, strain TS03 (*ΔsrrAB*)/ *Pvu*II.

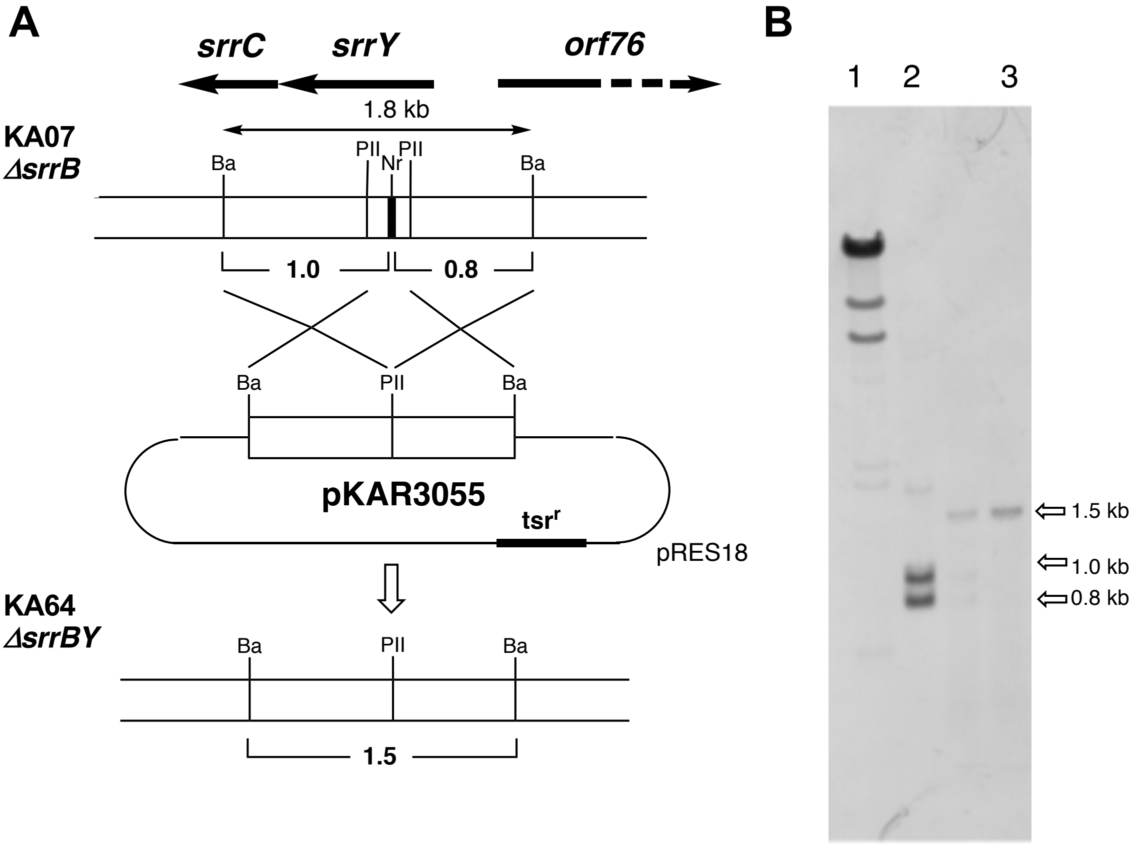

Figure S3. (**A**) Construction of the *ΔsrrBY* mutant KA64. Ba, *Bam*HI; Nr, *Nru*I; PII, *Pvu*II. (**B**) Southern blot analysis. Lane 1, λ/*Hin*dIII; lane 2, strain KA07 (parent)/ *Bam*HI & *Nru*I; lane 3, strain KA64 (*ΔsrrBY*)/ *Bam*HI & *Nru*I.

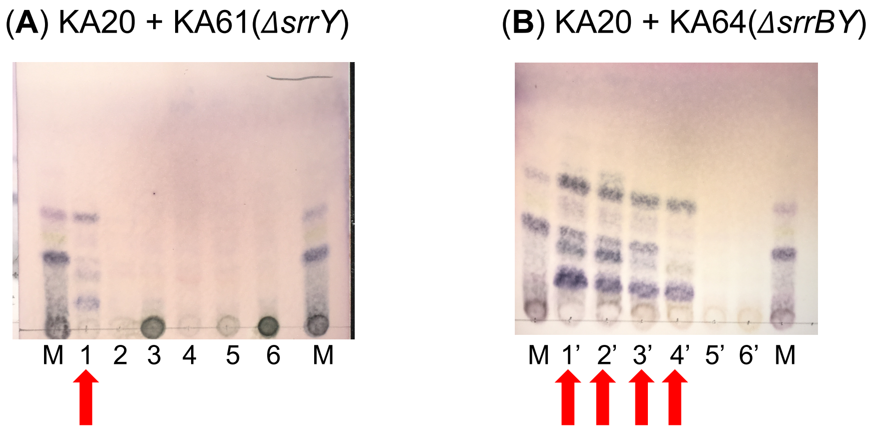

M; Standard sample **1** and **2**
1; Test strain KA20 (5 ml) + crude extracts of KA61 (5 ml culture equivalent) (1-fold)
2; Test strain KA20 (5 ml) + crude extracts of KA61 (3 ml culture equivalent) (0.6-fold)
3; Test strain KA20 (5 ml) + crude extracts of KA61 (1 ml culture equivalent) (0.2-fold)
4; Test strain KA20 (5 ml) + crude extracts of KA61 (0.6 ml culture equivalent) (0.12-fold)
5; Test strain KA20 (5 ml) + crude extracts of KA61 (0.3 ml culture equivalent) (0.06-fold)
6; Test strain KA20 (5 ml) + crude extracts of KA61 (0.25 ml culture equivalent) (0.05-fold)

M; Standard sample **1** and **2**
1’; Test strain KA20 (5 ml) + crude extracts of KA64 (5 ml culture equivalent) (1-fold)
2’; Test strain KA20 (5 ml) + crude extracts of KA64 (3 ml culture equivalent) (0.6-fold)
3’; Test strain KA20 (5 ml) + crude extracts of KA64 (1 ml culture equivalent) (0.2-fold)
4’; Test strain KA20 (5 ml) + crude extracts of KA64 (0.6 ml culture equivalent) (0.12-fold)
5’; Test strain KA20 (5 ml) + crude extracts of KA64 (0.3 ml culture equivalent) (0.06-fold)
6’; Test strain KA20 (5 ml) + crude extracts of KA64 (0.25 ml culture equivalent) (0.05-fold)

Figure S4. Effect of *srrB* mutation on SRB productivity. SRB assay was carried out using strain KA20, a mutant of SRB biosynthesis gene *srrX*, as a test strain. This strain restores antibiotic production in the presence of SRB fraction. Two strains for SRB donors, KA61 (*ΔsrrY*) (**A**) and KA64 (*ΔsrrBsrrY*) (**B**), were cultured for 30 h, and then extracted with ethyl acetate to obtain culture extracts. Antibiotic production was detected in lanes 1 (for KA61) and 1’-4’ (for KA64). TLC was developed with $CHCl_3$-MeOH = 15:1 (v/v) and stained with anisaldehyde-H_2SO_4.

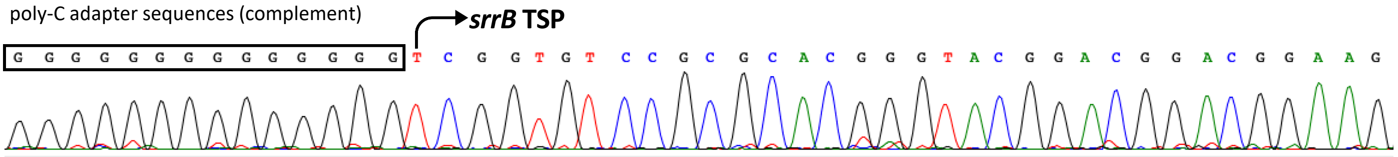

Figure S5. Determination of transcriptional start site (TSS) of *srrB* by 5' RACE system. Complement sequence of poly-C adaptor is indicated by a box. TSS of *srrB* is indicated by a bent arrow.

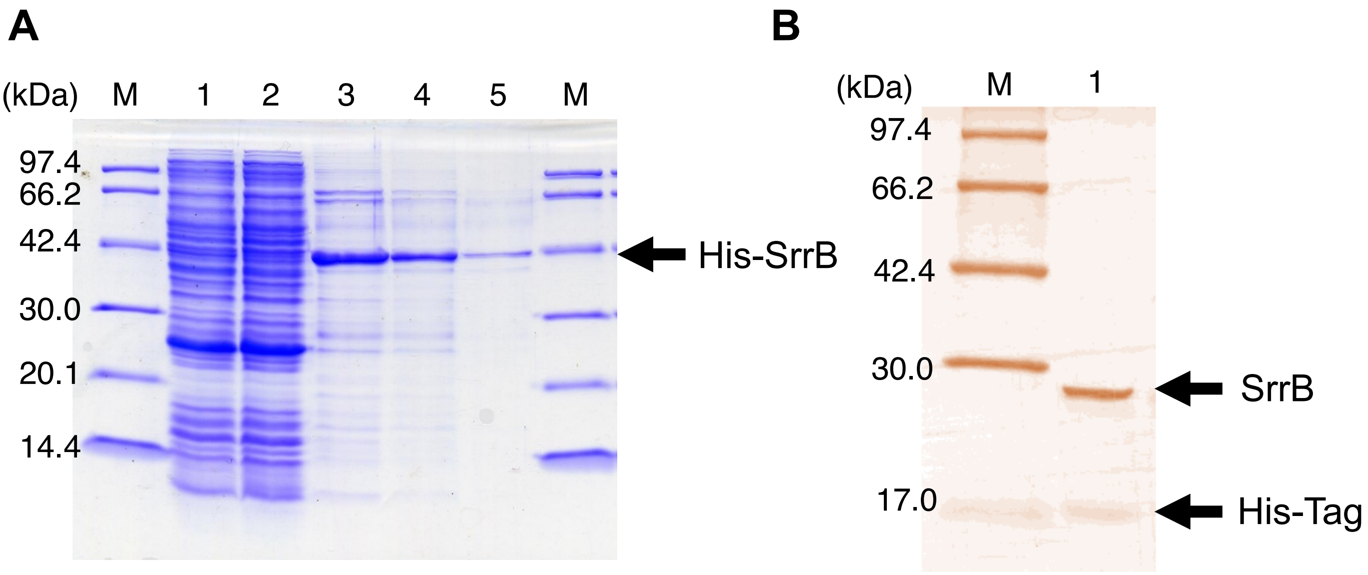

Figure S6. SDS-PAGE of SrrB protein expressed in *E. coli* BL21(DE3)pLysS.
(**A**) Purification of His-SrrB protein. Lane M, protein molecular weight markers; lanes 1 and 2, cell-free extract; lane 2, purified 41.0 kDa-sized His-SrrB prepared with the aid of a Ni^{2+}-nitrotriacetic acid agarose (Qiagen) (1st elution); lane 3. purified His-SrrB (2nd elution); purified His-SrrB (3rd elution). (**B**) Removal of His-Tag by enterokinase treatment. Lane M, protein molecular weight markers; lanes 1, purified SrrB digested with enterokinase. Marker sizes (kDa) are indicated on the left. All proteins were stained with Coomassie Brilliant Blue R250.
